# Identification of Potential Metabolites Mediating Bird's Selective Feeding on* Prunus mira* Flowers

**DOI:** 10.1155/2019/1395480

**Published:** 2019-06-23

**Authors:** Shanshan Zhang, Hong Ying, Gesang Pingcuo, Shuo Wang, Fan Zhao, Yongning Cui, Jian Shi, Hu Zeng, Xiuli Zeng

**Affiliations:** ^1^The Ministry of Agriculture of Qinghai-Tibet Plateau Fruit Trees Scientific Observation Test Station, Lhasa, Tibet 850032, China; ^2^Institute of Vegetables, Tibet Academy of Agricultural and Animal Husbandry Sciences, Lhasa, Tibet 850002, China; ^3^Wuhan Metware Biotechnology Co., Ltd, Wuhan 430070, China

## Abstract

In peach orchards, birds severely damage flowers during blossom season, decreasing the fruit yield potential. However, the wild peach species* Prunus mira* shows intraspecific variations of bird damage, indicating that some of the wild trees have developed strategies to avert bird foraging. Motivated by this observation, we formulated the present study to identify the potential flower metabolites mediating the bird's selective feeding behavior in* P. mira* flowers. The birds' preferred (FG) and avoided (BFT) flowers were collected from wild* P. mira* trees at three different locations, and their metabolite contents were detected, quantified, and compared. The widely-targeted metabolomics approach was employed to detect a diverse set of 603 compounds, predominantly, organic acids, amino acid derivatives, nucleotide and its derivatives, and flavones. By quantitatively comparing the metabolite contents between FG and BFT, three candidate metabolites, including Eriodictiol 6-C-hexoside 8-C-hexoside-O-hexoside, Luteolin O-hexosyl-O-hexosyl-O-hexoside, and Salvianolic acid A, were differentially accumulated and showed the same pattern across the three sampling locations. Distinctly, Salvianolic acid A was abundantly accumulated in FG but absent in BFT, implying that it may be the potential metabolite attracting birds in some* P. mira* flowers. Overall, this study sheds light on the diversity of the floral metabolome in* P. mira* and suggests that the bird's selective feeding behavior may be mediated by variations in floral metabolite contents.

## 1. Introduction

Bird damage is a persistent concern faced by fruit-growers, inflicting significant economic losses. Birds cause losses to horticulture by damaging or removing shoots, stems, foliage, flowers, and buds or fruits. In Australia, total bird damage to horticultural production was estimated at nearly $300 million annually [1]. Aggregate bird damage in five crops and states in the United States was estimated at $189 million [2]. More recently, Elser et al. [3] demonstrated that sweet cherry production of the United States decreased by about $185 to $238 million without the use of bird management. Unfortunately, the available techniques for bird damage management are mostly ineffective [2, 4].

Peach (*Prunus persica* (L.) Batsch) is the third most important of deciduous fruit trees worldwide and represents a model plant of Rosaceae family [5]. Despite its high economic value, peach like other fruit trees is significantly damaged by birds, particularly during the blossom season. Various types of birds feed on the flower's petals, which systematically drop off the tree together with the ovary, decreasing the fruit yield potential [1]. Unfortunately, a specific study has not yet been designed to evaluate the cost of this long-standing problem in peach orchards [6]. During our field visits, we observed that, unlike the cultivated peach, the wild peach trees (*Prunus mira* Koehne) display intraspecific variations of bird's visits and in the damage levels.* Turdus ruficollis* is the main bird species which damages* Prunus mira* flowers.* P. mira* is widely distributed along the Yarlung Zangbo Grand Canyon and its tributary basins in the Tibet plateau [7]. In fact, in very closely located wild trees, birds show preferences to some trees and only feed on flowers of these trees although there are no apparent differences in the flowers' phenotypes. This phenomenon, which we called “selective feeding behavior,” was observed in several locations. We inferred that the composition of the bird-preferred flowers may differ from the avoided ones.* P. mira* is itself an important economic fruit tree with medicinal values and has been proposed as an ancestral species of many cultivated peach species [8]. During domestication, crops typically experience population bottlenecks mainly due to an extensive artificial selection for improved quality and local adaptation [9]. Therefore, it is possible that wild peach species such as* P. mira* may have developed strategies to discourage bird's foraging, a mechanism absent in the cultivated peach due to the decline in diversity [10]. Similar observations were reported by Fonceka et al. [11] who demonstrated that large portions of useful alleles controlling important agronomic traits in wild species were left behind during peanut domestication.

Plants synthesize a staggering array of chemically diverse secondary metabolites, which have distinct biological functions, including immunity, pollinator attraction, defense against herbivory, etc. [12]. The inter- and intraspecific variations in the production of specialized metabolites have been widely observed and found to be largely genetically controlled [13]. In this study, we investigated the major discrepancies in the metabolic profiles of the preferred and avoided flowers from wild* P. mira* trees at three different locations in order to identify the potential metabolites mediating the bird's selective feeding behavior.

## 2. Results

### 2.1. Overview of the Metabolite Profiling in* Prunus mira* Flowers

**Figure 1:**
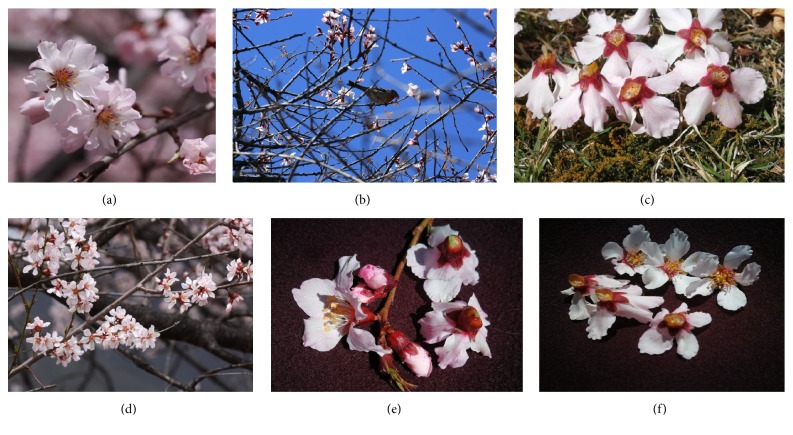
Photos of the* Prunus mira* flowers. (a) The bird-preferred flowers (FG) on the tree, (b)* Turdus ruficollis* feeding on* P. mira* flowers, (c) a high number of FG fed by birds dropped off the tree, (d) the avoided flowers (BFT) by birds on the tree, (e) wind which causes BFT flowers to drop off the tree but the ovary is intact, and (f) FG flowers with destroyed ovary.

**Figure 2:**
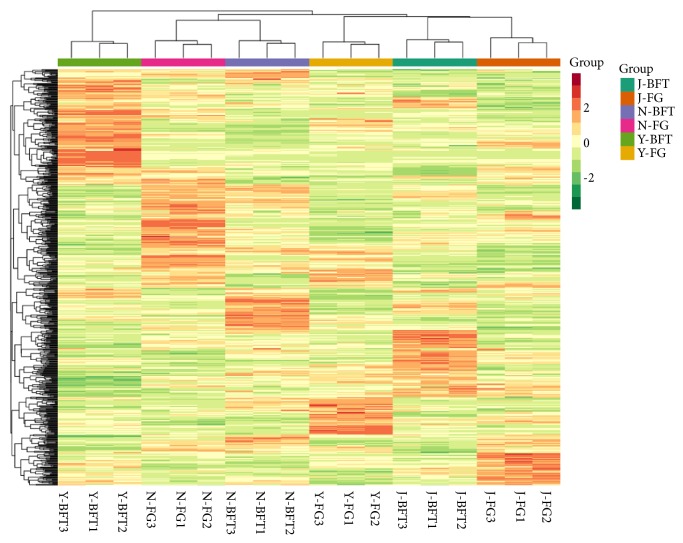
Heatmap clustering showing correlation among* Prunus mira* flower samples based on global metabolic profiles. Samples represent the preferred (FG) and avoided (BFT) flowers by birds collected at the J, N, and Y locations. Data represent the log2 fold change of the metabolite content.

Two types of* Prunus mira* flower samples, including the preferred (FG) and avoided (BFT) flowers, were collected at three different locations (“J,” “N,” and “Y”) (Figure 1). With three biological replicates, a total of 18 samples were used to portray the metabolic profiles employing the widely-targeted metabolomics approach. We successfully detected for the first time 603 compounds in* P. mira* flowers (Table S1). The metabolites detected in this work were diverse and rich and could be classified into 32 classes, predominantly, organic acids, amino acid derivatives, nucleotide and its derivatives, and flavone (Table 1). Very few compounds from the classes of pyridine derivatives, terpenoids, and flavonolignan were present in* P. mira* flowers. Based on the metabolite quantification, the samples were clustered using a heatmap hierarchical clustering approach. As shown in Figure 2, all the biological replicates were grouped together, indicating a good correlation between replicates and the high reliability of our data. The heatmap also showed that, while some metabolites were strongly accumulated in the flowers, others exist only in traces. In addition, contrasting patterns of metabolite content could be observed among FG and BFT, implying that the bird's selective feeding on flowers may be underpinned by the differential metabolite contents. Finally, the heatmap failed to group BFT and FG samples into two separate clades (Figure 2), suggesting that very few metabolites will likely distinguish the preferred and avoided flowers in* P. mira*.

### 2.2. Identifying the Differentially Accumulated Metabolites between FG and BFT

**Figure 3:**
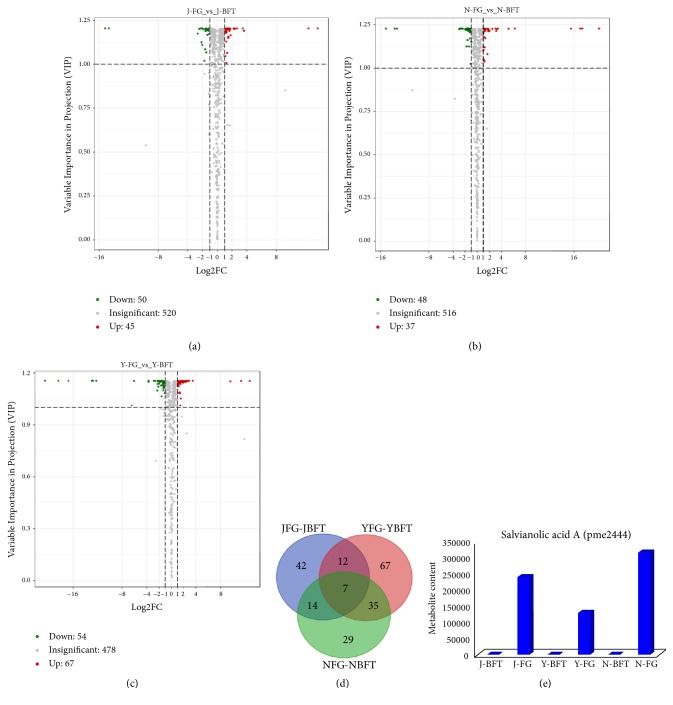
Identification of the potential metabolites associated with the bird's selective feeding behavior in* Prunus mira* flowers. (a) Volcano-plot showing the differentially accumulated metabolites (DAMs) between the preferred (FG) and avoided (BFT) flowers by birds at the J location (J-FG_vs_J-BFT), (b) volcano-plot showing the DAMs between the preferred (FG) and avoided (BFT) flowers by birds at the N location (N-FG_vs_N-BFT), (c) volcano-plot showing the DAMs between the preferred (FG) and avoided (BFT) flowers by birds at the Y location (Y-FG_vs_Y-BFT), (d) Venn diagram depicting the shared and unique DAMs between the three sampling locations, and (e) Salvianolic acid A content (pme2444) in FG and BFT samples collected at the three locations. DAMs were identified based on the variable importance in projection ≥ 1 and fold change ≥ 2 or fold change ≤ 0.5.

**Figure 4:**
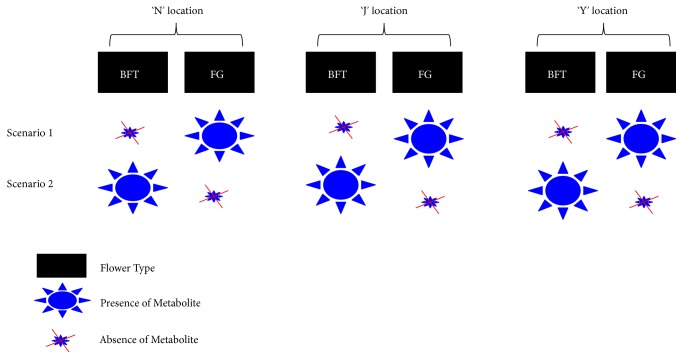
Schematic representation of the conceptual framework used in this study. Two scenarios were analyzed, in which the potential metabolites could be either present or absent in the preferred (FG) and avoided (BFT) flowers by birds. In either scenario, the same pattern for the metabolite differential accumulation between FG and BFT should be conserved across the three sampling locations (N, J, and Y).

We suspected that the variations in the metabolite contents of BFT and FG might be the leading reason of the bird's selective feeding on* P. mira* flowers. Therefore, we compared the flower metabolite profiles among FG and BFT samples. Metabolites with variable importance in projection (VIP) ≥ 1 and fold change ≥ 2 or fold change ≤ 0.5 were considered as differentially accumulated metabolites (DAM). At “J” location, 75 DAMs were identified for J-FG_vs_J-BFT, including 30 downaccumulated and 45 upaccumulated compounds in FG (Figure 3(a)). At “N” location, a similar number of DAMs (85) were detected for N-FG_vs_N-BFT, with 48 downaccumulated and 37 upaccumulated compounds in FG (Figure 3(b)). A conspicuously higher number of DAMs (121) were found at “Y” location for Y-FG_vs_Y-BFT, including 54 downaccumulated and 67 upaccumulated metabolites in FG (Figure 3(c)). Next, we compared the DAMs from the three locations and, interestingly, we found that seven metabolites were constitutively and differentially accumulated between FG and BFT, independently of the locations (Figure 3(d)). Of these seven metabolites, only three metabolites (pmb2954, pme2444, and pmb0619) conserved the same accumulation patterns between FG and BFT across the three locations and, therefore, fit in with our conceptual framework (see Materials and Methods, Figure 4, Table 2). These metabolites could be potentially associated with the bird's selective feeding on* P. mira* flowers. Distinctly, the metabolite pme2444 strongly fits in well with our conceptual framework. Pme2444 (Salvianolic acid A) was highly accumulated in FG but absent in BFT in all the three locations, corresponding to scenario 1 (Figure 3(e)).

## 3. Discussion

The destruction of flowers and buds by birds on fruit trees is a long-standing source of complaint by fruit-growers [14]. This leads to severe yield loss and economic damage for producers across the globe [2]. Observations by Bray et al. [15] denoted that birds concentrate their feeding activity in a particular area and ignore others. Further studies have also observed the selective feeding behavior of birds in agricultural crops [16, 17], suggesting the existence of some underlying biological factors. In fact, plants have developed different mechanisms to reduce or avoid enemies, including specific responses that activate different metabolic pathways, which considerably alter their chemical and physical aspects [18]. Long-term interactions with their enemies have sculpted plant metabolism, resulting in a natural variation in metabolites that control important ecological and agronomic traits such as resistance to pests [19].

In the particular case of peach orchards, birds significantly damage the flowers by feeding on the petals. In contrast to the cultivated peach, we observed that wild peach (*Prunus mira*) exhibited an intraspecific variation of bird's visit, showing that some of the wild trees have developed strategies to discourage bird forage. Bao et al. [8] reported a high genetic differentiation among wild* Prunus mira* populations, which could be translated into a high metabolic diversity. This was confirmed by the great variation in the metabolite contents of the sampled flowers from different wild trees in our study. By comparing the metabolite profiles of the preferred and avoided flowers, we pinpointed three candidate compounds (Eriodictiol 6-C-hexoside 8-C-hexoside-O-hexoside, Luteolin O-hexosyl-O-hexosyl-O-hexoside, and Salvianolic acid A), which were differentially accumulated in the two types of flowers, independently of the sampling locations. We speculate that the variation of flower metabolites among* P. mira* trees could be an adaptation mechanism to avoid bird's damage. Both Eriodictiol and Luteolin are flavonoid related compounds and were found significantly accumulated in the avoided flower samples. These compounds have been established as antioxidant and anti-inflammatory agents [20–22]. Although flavonoid metabolites are known to be involved in plant defense [23], whether Eriodictiol and Luteolin act as deterrent agents in* P. mira* flowers against birds is still unknown and will require further investigations. Conversely, Salvianolic acid A was strongly accumulated in the preferred flowers but absent in the avoided ones, implying that birds might be principally attracted to* P. mira* flower-containing Salvianolic acid A. Hence, it is tempting to speculate that the impaired production of Salvianolic acid A in BFT could be a defense strategy to avoid bird's visits. Recent lines of evidence indicated that Salvianolic acid A is connected with the MAPK pathways and attenuates oxidative stress in human [24, 25], but how and why this molecule may attract birds is still unclear. The present study is the first attempt to clarify the bird's selective feeding behavior in* P. mira* flowers. Given the importance of the subject and the potential of our findings, future investigations are needed. To consolidate our results, we plan to extend the sampling area and compare the identified candidate metabolites between more* P. mira* trees and also in various cultivated peach genotypes. Moreover, other wild* Prunus* species will be investigated to assess whether the phenomenon is common in the wild related species. A deep understanding of the biological activity of these metabolites will help formulate sustainable strategies for a better protection of peach orchards against bird's flower damage.

## 4. Materials and Methods

### 4.1. Study Area and Flower Sampling

The study was conducted in the Milin County, Nyingchi City, Tibet Autonomous Region, China (29°38′12′′N latitude, 94°21′40′′E longitude). Samples were collected in March during blossom season at three different locations, namely, “J,” “N,” and “Y”, each separated by 15 km in average. At each location, we targeted two close wild* Prunus mira* trees, which have contrasting bird's visits. The highly visited trees by birds can be clearly distinguished by the significant numbers of fallen flowers containing the ovary (FG) underneath (Figures 1(a) and 1(b)). We named as BFT the flowers from the avoided trees (Figure 1(c)). FG and BFT samples were collected on the respective trees at the same period. The samples were composed of the entire corolla, including the ovary. Approximately, 10-15 flowers from three random parts of the same tree were considered as biological replicates. In total, 18 samples were collected, frozen immediately in liquid nitrogen in the field, transported to the laboratory, and then stored at −80°C until further use.

### 4.2. Metabolic Profiling

The sample preparation, extract analysis, metabolite identification, and quantification were performed at Wuhan MetWare Biotechnology Co., Ltd. (www.metware.cn), following their standard procedures and previously described by Yuan et al. [26].

### 4.3. Sample Preparation and Extraction

The frozen samples were crushed using a mixer mill (MM 400, Retsch) with a zirconia bead for 1.5 min at 30 Hz. About 100 mg powder was weighted and extracted overnight at 4°C with 1 ml 70% aqueous methanol. Following centrifugation at 10,000 g for 10 min, the extracts were absorbed (CNWBOND Carbon-GCB SPE Cartridge, 250 mg, 3 ml; ANPEL, Shanghai, China, www.anpel.com.cn/cnw) and filtrated (SCAA-104, 0.22 *μ*m pore size; ANPEL, Shanghai, China, http://www.anpel.com.cn/) before LC-MS analysis [27].

### 4.4. HPLC Conditions

The sample extracts were analyzed using an LC-ESI-MS/MS system (HPLC, Shim-pack UFLC SHIMADZU CBM30A system, www.shimadzu.com.cn/; MS, Applied Biosystems 6500 Q TRAP, www.appliedbiosystems.com.cn/). The analytical conditions were as follows: HPLC: column, Waters ACQUITY UPLC HSS T3 C18 (1.8 *μ*m, 2.1 mm*∗*100 mm); solvent system, water (0.04% acetic acid): acetonitrile (0.04% acetic acid); gradient program, 100:0V/V at 0 min, 5:95V/V at 11 min, 5:95V/V at 12 min, 95:5V/V at 12.1 min, 95:5V/V at 15 min; flow rate, 0.40 ml/min; temperature, 40°C; injection volume: 2 *μ*l. The effluent was alternatively connected to an ESI-triple quadrupole-linear ion trap (Q TRAP)-MS.

### 4.5. ESI-Q TRAP-MS/MS

Linear ion trap (LIT) and triple quadrupole (QQQ) scans were acquired on a triple quadrupole-linear ion trap mass spectrometer (Q TRAP), API 6500 Q TRAP LC/MS/MS System, equipped with an ESI Turbo Ion-Spray interface, operating in a positive ion mode and controlled by Analyst 1.6 software (AB Sciex). The ESI source operation parameters were as follows: ion source, turbo spray; source temperature 500°C; ion spray voltage (IS) 5500 V; ion source gas I (GSI), gas II (GSII), and curtain gas (CUR) were set at 55, 60, and 25 psi, respectively; the collision gas (CAD) was high. Instrument tuning and mass calibration were performed with 10 and 100 *μ*mol/L polypropylene glycol solutions in QQQ and LIT modes, respectively. Based on the self-built database MetWare Database (http://www.metware.cn/) and metabolite information in public database, the materials were qualitatively analyzed according to the secondary spectrum information and the isotope signal was removed during the analysis. QQQ scans were acquired as multiple reaction monitoring (MRM) experiments with collision gas (nitrogen) set to 5 psi [28]. Declustering potential (DP) and collision energy (CE) for individual MRM transitions were done with further DP and CE optimization [27]. A specific set of MRM transitions were monitored for each period according to the metabolites eluted within this period.

### 4.6. Metabolite Data Analysis

Before the data analysis, quality control (QC) analysis was conducted to confirm the reliability of the data. The QC sample was prepared by the mixture of sample extracts and inserted into every two samples to monitor the changes in repeated analyses. Data matrices with the intensity of the metabolite features from the 18 samples were uploaded to the Analyst 1.6.1 software (AB SCIEX, Ontario, Canada) for statistical analyses. The supervised multivariate method, partial least squares-discriminant analysis (PLS-DA), was used to maximize the metabolome differences between the two flower samples. The relative importance of each metabolite to the PLS-DA model was checked using the parameter called variable importance in projection (VIP). Metabolites with VIP ≥ 1 and fold change ≥ 2 or fold change ≤ 0.5 were considered as differential metabolites for group discrimination [26]. Heatmap based on the hierarchical cluster analysis method was performed in the R software (www.r-project.org).

### 4.7. Conceptual Framework

Two different types of flower samples (BFT and FG) were collected from different trees at three locations. Given that birds mainly prefer FG over BFT, we postulated that the main difference between these two samples might be related to the presence/absence pattern or at least a large discrepancy in the quantity of one or several key metabolites. Two scenarios are therefore possible: (1) birds are attracted by some key flower metabolites, and, then, these molecules should be highly accumulated in FG but absent or only present in trace in BFT; (2) birds are repelled by some key flower metabolites, and, then, these molecules should be highly accumulated in BFT but absent or only present in trace in FG (Figure 4). The flower metabolites, whose accumulation patterns fit in with either of these two scenarios and consistently across the three locations, were regarded as the candidate molecules governing bird's selective feeding on* Prunus mira* flowers (Figure 4).

## Figures and Tables

**Table 1:** Classification of the 603 detected metabolites into major classes.

Class	Number of compounds	Class	Number of compounds
Organic acids	68	Anthocyanins	13
Amino acid derivatives	53	Lipids_Glycerolipids	13
Nucleotide and its derivates	52	Vitamins	12
Flavone	42	Catechin derivatives	11
Flavonol	35	Phenolamides	10
Lipids_Glycerophospholipids	32	Isoflavone	10
Hydroxycinnamoyl derivatives	28	Indole derivatives	7
Others	27	Alcohols and polyols	7
Amino acids	26	Cholines	5
Flavone C-glycosides	24	Tryptamine derivatives	5
Quinate and its derivatives	22	Proanthocyanidins	5
Coumarins	18	Nicotinic acid derivatives	3
Carbohydrates	18	Alkaloids	3
Lipids_Fatty acids	17	Pyridine derivatives	2
Flavanone	17	Terpenoids	1
Benzoic acid derivatives	16	Flavonolignan	1

**Table 2:** List of the seven metabolites differentially expressed between FG and BFT and conserved across the three sampling locations (J, N, and Y).

ID	Name	Class	Log2 Fold Change		
			JFG-JBFT	YFG-YBFT	NFG-NBFT
pmb0848	LysoPC 16:1 (2n isomer)	Lipids_Glycerophospholipids	1.11	2.79	-1.39
pmb0863	LysoPC 16:2 (2n isomer)	Lipids_Glycerophospholipids	1.58	2.87	-1.53
pmb0865	LysoPC 18:3 (2n isomer)	Lipids_Glycerophospholipids	1.32	1.76	-1.02
pmb2228	LysoPC 19:0	Lipids_Glycerophospholipids	1.23	1.95	-1.60
pmb2954	Luteolin O-hexosyl-O-hexosyl-O-hexoside	Flavone	2.30	1.15	1.24
pme2444	Salvianolic acid A	Other	-Inf*∗*	-Inf*∗*	-Inf*∗*
pmb0619	Eriodictiol 6-C-hexoside 8-C-hexoside-O-hexoside	Flavone C-glycosides	2.41	1.03	1.29

*∗* The metabolite was not detected in the BFT samples.

## Data Availability

The data used to support the findings of this study are available from the corresponding author upon request.

## Supplementary Materials

Supplementary Materials Table S1: overview of the metabolites detected and quantified in two types of* Prunus mira* samples: preferred (FG) and avoided (BFT) flowers by birds at three different locations (J, N, and Y). Data are from three biological replicates and the mix01 to mix08 represent the mixture of sample extracts for quality check.
